# Antibodies elicited by mRNA-1273 vaccination bind more broadly to the receptor binding domain than do those from SARS-CoV-2 infection

**DOI:** 10.1126/scitranslmed.abi9915

**Published:** 2021-06-30

**Authors:** Allison J. Greaney, Andrea N. Loes, Lauren E. Gentles, Katharine H.D. Crawford, Tyler N. Starr, Keara D. Malone, Helen Y. Chu, Jesse D. Bloom

**Affiliations:** 1 Basic Sciences Division and Computational Biology Program, Fred Hutchinson Cancer Research Center, Seattle, WA 98109, USA.; 2 Department of Genome Sciences & Medical Scientist Training Program, University of Washington, Seattle, WA 98195, USA.; 3 Howard Hughes Medical Institute, Chevy Chase, MD 20815, USA.; 4 Department of Microbiology, University of Washington, Seattle, WA 98195, USA.; 5 Division of Allergy and Infectious Diseases, University of Washington, Seattle, WA 98195, USA.

## Abstract

The emergence of severe acute respiratory syndrome coronavirus 2 (SARS-CoV-2) variants with mutations in key antibody epitopes has raised concerns that antigenic evolution could erode adaptive immunity elicited by prior infection or vaccination. The susceptibility of immunity to viral evolution is shaped in part by the breadth of epitopes targeted by antibodies elicited by vaccination or natural infection. To investigate how human antibody responses to vaccines are influenced by viral mutations, we used deep mutational scanning to compare the specificity of polyclonal antibodies elicited by either two doses of the mRNA-1273 COVID-19 vaccine or natural infection with SARS-CoV-2. The neutralizing activity of vaccine-elicited antibodies was more targeted to the receptor-binding domain (RBD) of the SARS-CoV-2 spike protein compared to antibodies elicited by natural infection. However, within the RBD, binding of vaccine-elicited antibodies was more broadly distributed across epitopes compared to infection-elicited antibodies. This greater binding breadth means that single RBD mutations have less impact on neutralization by vaccine sera compared to convalescent sera. Therefore, antibody immunity acquired by natural infection or different modes of vaccination may have a differing susceptibility to erosion by SARS-CoV-2 evolution.

## Binding Breadth

Antibody responses elicited by natural infection or vaccination against the same target are known to differ. However, the extent to which antibody responses to the SARS-CoV-2 receptor binding domain (RBD) differ between vaccination and infection has not been fully characterized. Here, Greaney *et al.* demonstrated that neutralizing antibodies elicited by immunization with the Moderna mRNA-1273 vaccine were more focused to the RBD than those elicited by natural infection. However, vaccination-elicited antibodies targeted a broader range of epitopes within the RBD than infection-elicited antibodies. These findings demonstrate that the type of exposure, including different types of vaccines or infection, can influence the antibody response to SARS-CoV-2.

## Teaser

Deep mutational scanning shows that the mRNA-1273 RBD-binding antibody response is less affected by single viral mutations than the infection response.

## INTRODUCTION

Mitigation of the coronavirus disease 2019 (COVID-19) pandemic will depend on population immunity acquired via infection with or vaccination against severe acute respiratory syndrome coronavirus 2 (SARS-CoV-2). Unfortunately, humans are repeatedly reinfected with the endemic “common-cold” coronaviruses (*1*), at least in part because these viruses evolve to escape neutralizing antibody immunity elicited by prior infection (*2*). SARS-CoV-2 is already undergoing similar antigenic evolution, with the recent emergence of new viral lineages with reduced neutralization by antibodies elicited by infection and vaccination (*3*–*8*). Preliminary results suggest that immunity still provides substantial protection against infection and severe disease (*9*, *10*) caused by these new viral lineages; however, if SARS-CoV-2 is similar to other human coronaviruses, then, at minimum, the protection against reinfection will eventually be eroded by viral evolution.

However, unlike other human coronaviruses, a large fraction of the population is acquiring SARS-CoV-2 immunity from vaccination rather than infection. The first two vaccines approved for emergency use in the United States were Moderna’s mRNA-1273 and Pfizer/BioNTech’s BNT162b2. Both mRNA vaccines encode the full SARS-CoV-2 spike ectodomain with a transmembrane anchor and stabilizing S-2P mutations (*11*). It is possible that these vaccines could elicit antibodies with distinct specificities compared to natural infection due to variation in the spike (such as the S-2P mutations) or divergent immune responses to a two-dose mRNA vaccine versus infection. If the specificities differ, this could influence the impact of viral evolution on SARS-CoV-2 immunity.

To address this question, we used a combination of serological assays and deep mutational scanning to map the specificity of the human polyclonal antibody response after two doses of the mRNA-1273 vaccine. The vaccine elicited neutralizing activity that is even more targeted to the spike receptor-binding domain (RBD) than infection-elicited immunity. However, within the RBD, binding by vaccine-elicited antibodies was often less affected by single mutations. As a result, common RBD mutations sometimes eliminated less of the neutralizing activity of mRNA-1273 vaccine sera than convalescent sera, and vaccine sera retained substantial RBD-directed neutralization even in the presence of mutations to three major RBD neutralizing epitopes.

## RESULTS

### The neutralizing activity of mRNA-1273 vaccine–elicited antibodies is more RBD-targeted than that of infection-elicited antibodies

We studied sera from adults (ages 18 to 55 years) who received two doses of the Moderna mRNA-1273 vaccine in phase 1 clinical trials (*12*). The majority of our study focused on 14 individuals who received the 250-μg dose, although we validated key conclusions with a smaller subset of eight trial participants who received the 100-μg dose. The sera were collected at 36 and 119 days after the first vaccine dose, corresponding to 7 and 90 days after the second dose. It was previously shown that these individuals had high amounts of binding and neutralizing antibodies against SARS-CoV-2, with neutralizing antibody titers within the upper quartile of sera from SARS-CoV-2 convalescent individuals (*12*). Throughout, we compared vaccine sera to convalescent plasma or serum samples from two independent cohorts (*13*, *14*). The convalescent plasma samples were characterized in earlier studies (*13*–*16*) and grouped into an early time point of 15 to 60 days after symptom onset and a late time point of 100 to 150 days after symptom onset.

**Fig. 1:**
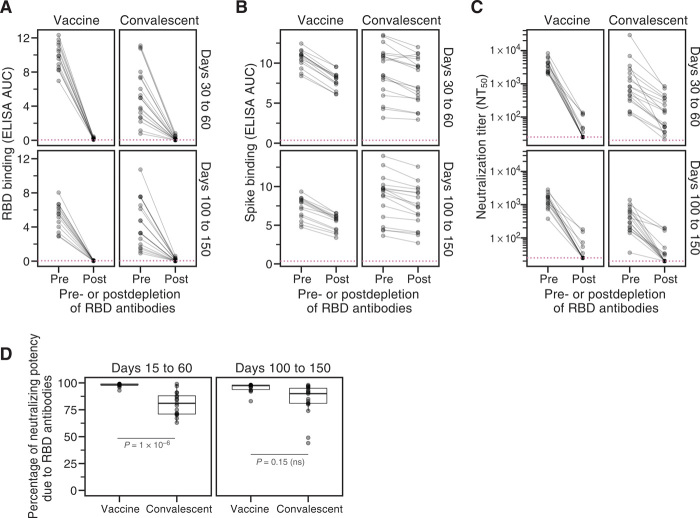
RBD-binding antibodies are responsible for most neutralizing activity of mRNA-1273 vaccine–elicited sera. (**A**) Binding of serum antibodies to SARS-CoV-2 RBD, as measured by ELISA area under the curve (AUC), for vaccine-elicited sera and convalescent plasma before and after depletion of RBD-binding antibodies. The dashed pink line indicates binding of prepandemic sera. (**B**) Binding of serum antibodies to the full spike ectodomain. The *y*-axis scale units in (A) and (B) are not comparable between samples from vaccinated and convalescent individuals owing to different dilution factors (beginning at 1:500 for vaccine sera and 1:100 for convalescent plasma samples). (**C**) Neutralization titer (NT_50_) of vaccine-elicited sera and convalescent plasma samples before and after depletion of RBD-binding antibodies. The limit of detection is shown as a dashed horizontal pink line. (**D**) Percentage of neutralizing activity of vaccine-elicited sera and convalescent plasma samples due to RBD-binding antibodies. *P* values are from a log-rank test accounting for censoring. *n* = 17 for each time point for convalescent plasma samples and *n* = 14 for each time point for vaccine sera. ns, not significant. All measurements of convalescent plasma binding and neutralization were previously reported in (*15*).

The majority of the neutralizing activity of convalescent sera and plasma is due to RBD-binding antibodies (*15*, *17*, *18*). To determine whether neutralization by vaccine sera is similarly RBD-targeted, we depleted RBD-binding antibodies from the day 36 and 119 sera isolated from 14 individuals who received the 250-μg dose of the mRNA-1273 vaccine. We then measured serum immunoglobulin G (IgG) binding to the RBD and full spike ectodomain before and after depletion. As expected, depletion removed all RBD-binding antibodies (Fig. 1A and fig. S1, A and B). However, depleting RBD-binding antibodies only moderately decreased spike-binding activity in either vaccine sera or convalescent plasma (Fig. 1B and fig. S1B), consistent with studies showing that a minority of spike-binding vaccine-elicited B cells target the RBD (*5*, *19*).

To determine the contribution of RBD-binding antibodies to neutralization, we measured the neutralizing activity of vaccine sera before and after depleting RBD-binding antibodies using spike-pseudotyped lentiviral particles. For samples isolated from 13 of 14 vaccinated individuals, greater than 90% of the neutralizing activity at both time points was dependent on RBD-binding antibodies (Fig. 1, C and D, and data file S1). For 17 of 28 vaccine sera, depletion of RBD-binding antibodies reduced the neutralization titer (reciprocal IC_50_) from >1000 to <25 (Fig. 1, C and D, and fig. S1, C and D). The percentage of neutralizing activity due to RBD-binding antibodies was higher for vaccine sera than for convalescent plasma samples collected between days 15 and 60 (*P* = 1.0 × 10^−6^; Fig. 1, C and D) (*15*). These assays were performed in 293T cells overexpressing human angiotensin-converting enzyme 2 (ACE2), which may underestimate contributions of non–RBD-binding antibodies to viral neutralization (*6*, *20*, *21*). Nonetheless, because the same assay was used for vaccine and convalescent samples, we conclude that the neutralizing activity of the antibody response elicited by the mRNA-1273 vaccine is more targeted to the RBD than for infection-elicited antibodies.

### Complete mapping of RBD mutations that reduce binding by vaccine-elicited sera at 119 days after vaccination reveals broad binding specificity across multiple RBD epitopes

**Fig. 2:**
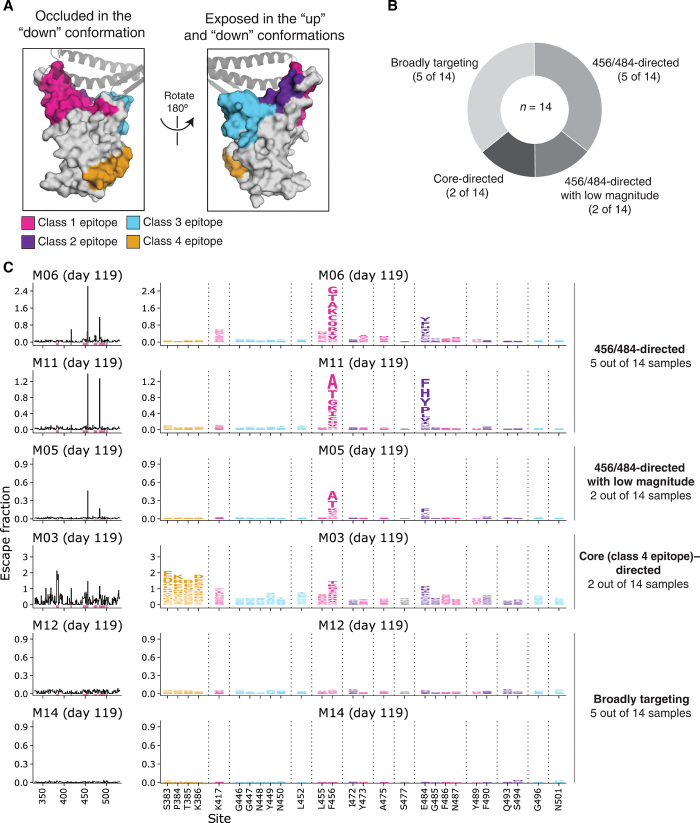
Complete maps of RBD mutations that reduce binding by sera collected 119 days after vaccination with the 250-μg dose of the mRNA-1273 vaccine. (**A**) The epitopes of four major classes (*24*) of RBD-binding antibodies are colored on the RBD surface (PDB 6M0J). ACE2 is shown as a gray ribbon diagram. (**B**) Number of sera that fell into each of the four major categories of binding-escape maps as categorized by subjective visual inspection. (**C**) Escape maps for six representative sera are shown. The line plots on the left indicate the sum of effects of all mutations at each RBD site on serum antibody binding, with larger values indicating more escape. The logo plots on the right show key sites (highlighted in purple on the line plot *x* axes). The height of each letter is that mutation’s escape fraction; larger letters indicate a greater reduction in binding. Escape fractions are not strictly comparable between samples owing to the use of sample-specific FACS selection gates; therefore, for each sample, the *y* axis is scaled independently. RBD sites are colored by epitope as in (A). The escape fractions were correlated between independent libraries, and we report the average of duplicate measurements throughout. Interactive versions of logo plots and structural visualizations are at https://jbloomlab.github.io/SARS-CoV-2-RBD_MAP_Moderna/.

We used deep mutational scanning (*15*, *22*) to map all mutations to yeast-displayed RBD that reduced vaccine serum antibody binding. Our experiments used duplicate libraries containing 3804 of the 3819 possible single amino acid mutations to the RBD of the Wuhan-Hu-1 strain of SARS-CoV-2, 2034 of which are tolerated for proper protein folding and at least modest ACE2 binding (*23*). We incubated the yeast-displayed libraries with each serum and used fluorescence-activated cell sorting (FACS) to enrich for the 3 to 5% of cells expressing RBD mutants with the lowest amount of serum binding (figs. S2 and S3 and table S1). The degree to which mutations reduce serum binding varies across samples, so the FACS gates were set separately for each sample. We used deep sequencing to quantify the “escape fraction” for each of the 2034 tolerated RBD mutations against each serum by determining the frequency of each mutant in the serum-escape bin versus the original unsorted population. These escape fractions range from 0 (no cells with the mutation in the serum-escape bin) to 1 (all cells with the mutation in the serum-escape bin) (data file S2). Correlations between escape fractions measured for independent biological replicate libraries are shown in fig. S4. We represent the escape maps as logo plots, where the height of each letter is proportional to its escape fraction (Fig. 2 and figs. S5 and S6).

The escape maps for sera collected at day 119 from individuals who received the 250-μg vaccine dose fell into four qualitative categories (Fig. 2, A and B) (*24*). For 5 of 14 individuals, escape from antibody binding was focused on RBD sites 456 and 484 (Fig. 2, B and C, and fig. S5). These two sites are on the receptor-binding ridge in the neutralizing “class 1” and “class 2” RBD epitopes, respectively (Fig. 2A) (*24*). Two more individuals also had escape maps that were focused on sites 456 and 484 but with a very low overall magnitude of escape (Fig. 2, B and C, and fig. S5). For 2 of 14 individuals, serum binding was most affected by mutations in the “class 4” epitope located in the core RBD, including sites 383 to 386 (Fig. 2 and fig. S5). Antibodies targeting the class 4 epitope are often non-neutralizing or less potently neutralizing than antibodies targeting the receptor-binding motif (*17*, *18*, *25*, *26*). The escape maps for the remaining five individuals were “flat,” meaning that no single mutation had a large effect on serum binding, suggestive of broad binding to multiple RBD epitopes (Fig. 2, B and C, and fig. S5).

To determine whether the vaccine dose affected the RBD-binding specificity of the polyclonal antibody response, we mapped binding escape from the day 119 sera from eight individuals vaccinated with 100-μg rather than 250-μg doses. The escape maps of the 100-μg cohort resembled those of the 250-μg cohort and fell into the 456/484-targeting, core-targeting, or flat categories (fig. S6). Although the sample sizes are small, and a higher fraction of the 100-μg dose escape maps were flat than for the 250-μg cohort (4 of 8 versus 5 of 14, respectively), this suggests that 100- and 250-μg doses elicit antibody responses similar in the breadth of their RBD-binding specificity.

### Binding-escape maps become more targeted to specific sites in the RBD from 36 to 119 days after vaccination

**Fig. 3:**
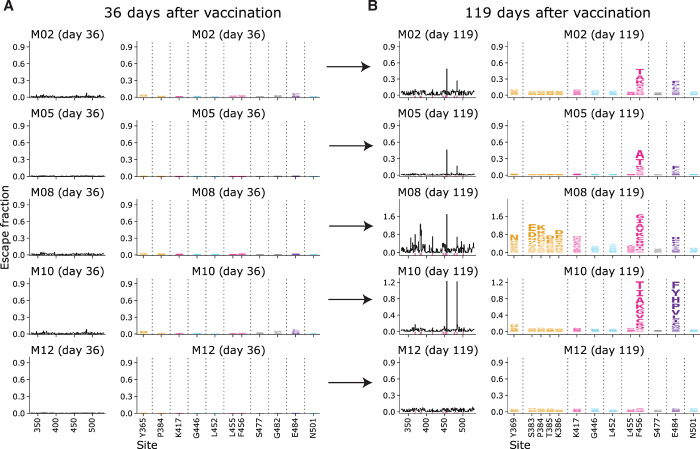
Comparison of escape maps for sera collected at days 36 and 119 after vaccination shows that the RBD-binding response becomes more focused over time. (**A** and **B**) Escape maps for sera at day 36 (A) and day 119 (B) from five individuals who received the 250-μg vaccine dose are shown. The day 36 maps are all relatively flat, indicating that no RBD mutation has a large effect on serum antibody binding. By day 119, the maps are often more focused on sites 456 and 484. The *y* axis is scaled separately for each serum sample. Interactive versions are at https://jbloomlab.github.io/SARS-CoV-2-RBD_MAP_Moderna/.

To examine longitudinal changes in binding specificity of vaccine-elicited serum antibodies to the RBD, we also determined binding-escape maps for sera collected at day 36 after vaccination from five individuals who received the 250-μg dose (Fig. 3). All of these day 36 sera had relatively flat escape maps, meaning that no single mutation had a large effect on serum binding (Fig. 3A). However, by day 119, the escape maps for most individuals were more focused on specific sites in the RBD (Fig. 3B). Specifically, for four of five individuals, the escape maps became focused on RBD sites 456 and 484 (Fig. 3B). For one of these individuals, the focusing on sites 456 and 484 was accompanied by increased focusing on the class 4 epitope, including sites 383 to 386. Only one individual, M12, had a day 119 escape map as flat as the day 36 escape map. These results suggest that, as the vaccine-induced RBD-binding antibody response matures over time, it becomes more focused on specific sites in the RBD.

### RBD binding by vaccine-elicited serum samples is broader than for convalescent plasma samples

**Fig. 4:**
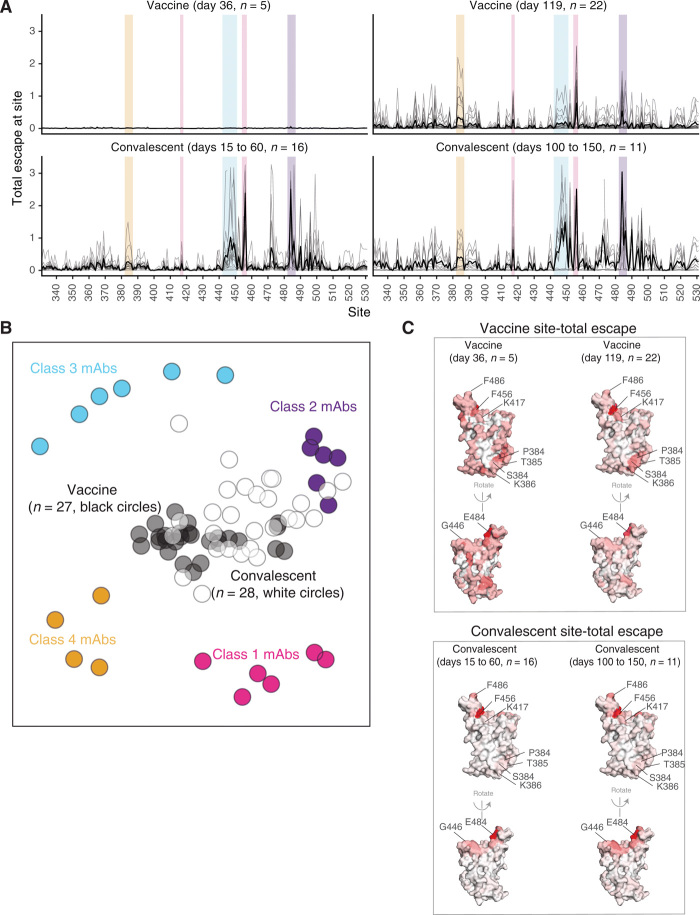
The binding of vaccine-elicited polyclonal antibodies is more broadly distributed across the RBD than the binding of infection-elicited antibodies. (**A**) Escape from RBD-binding antibodies at each site in the RBD was mapped for vaccine sera or convalescent plasma samples collected at early or late time points. Thin gray lines show individual serum or plasma samples, and the thick black line shows the mean (number of samples is indicated in the plot titles). Key sites within the epitopes of each major RBD antibody class are highlighted with the colors defined in Fig. 2A and in (B). (**B**) Relationships among escape maps of vaccine sera, convalescent plasma samples, and monoclonal antibodies visualized with a multidimensional scaling projection. Vaccine sera include both doses and time points. Convalescent plasma samples include all time points. (**C**) Total binding escape at each site mapped onto the RBD surface after averaging across all serum or plasma in each group. The RBD surface coloring is scaled from white to red, with white indicating no escape, and red indicating the site with the greatest escape. The color scaling spans the full range of white to red for each serum or plasma group, so a quantitative scale is not comparable across groups. Escape maps for monoclonal antibodies are previously described in (*16*, *22*, *27*–*29*), and convalescent plasma samples are described in (*15*, *16*). An interactive version of (B) where you can mouse over points for details is at https://jbloomlab.github.io/SARS-CoV-2-RBD_MAP_Moderna/mds.html.

To elucidate differences in the specificity of the RBD-binding antibody response elicited by vaccination versus infection, we compared the vaccine-sera escape maps to ones that we previously determined for convalescent plasma samples (*15*, *16*). At both 15- to 60-day and 100- to 150-day ranges, the convalescent escape maps were more focused on specific RBD sites than the vaccine escape maps (Fig. 4A). The difference was especially notable at the early time point, where the day 36 vaccine samples all had flat escape maps, whereas the convalescent samples often had escape maps indicating that antibody binding was strongly affected by mutations at specific RBD sites such as 456 and 484 (Fig. 4A). The difference between the vaccine and convalescent samples was less notable at the later time point, but the convalescent maps were still more focused than the vaccine maps, as demonstrated by the lower magnitude of the escape fractions. There were also differences in the RBD sites where mutations affected binding for the vaccine versus convalescent samples. Although most samples of both types were affected by mutations at sites 456 and 484, the convalescent samples tended to also be affected by mutations to the 443-450 loop in the class 3 epitope, whereas mutations in the class 4 epitope spanning sites 383 to 386 sometimes had a more pronounced effect on the vaccine samples (Figs. 2 and 4A and fig. S5).

To visualize relationships between vaccine- and infection-elicited antibody responses, we used multidimensional scaling to create a two-dimensional projection of the escape maps for the vaccine serum samples, convalescent plasma samples (*15*, *16*), and previously characterized monoclonal antibodies (Fig. 4B; an interactive version where you can mouse over points for details is at https://jbloomlab.github.io/SARS-CoV-2-RBD_MAP_Moderna/mds.html) (*16*, *22*, *27*–*29*). In this projection, monoclonal antibodies, sera samples, or plasma samples with similar binding-escape mutations are located close together, whereas those affected by distinct mutations are far apart. As previously reported (*16*), convalescent plasma samples clustered closest to class 2 antibodies (Fig. 4B), which are generally most affected by mutations to site 484. In contrast, the vaccine sera were more centrally located in the middle of the antibodies of all four classes, reflecting their flatter binding-escape maps that were less dominated by mutations that escape any single antibody class (Fig. 4B).

To examine sites of binding-escape mutations in the context of the RBD’s structure, we projected the total escape at each site averaged across all vaccine or convalescent samples at each time point onto the surface of the RBD (Fig. 4C). The sites where mutations affected binding of vaccine sera were broadly distributed across the RBD surface (Fig. 4C), whereas convalescent plasma samples were most affected by mutations at just a few key regions (sites 456 and 484 and, to a lesser degree, the 443-450 loop) (Fig. 4C). However, as noted above, binding escape from the vaccine sera was somewhat more focused at day 119 relative to day 36, including at sites 456, 484, and 383 to 386.

### Single RBD mutations have less impact on vaccine-elicited antibody neutralizing activity than infection-elicited antibody neutralizing activity

**Fig. 5:**
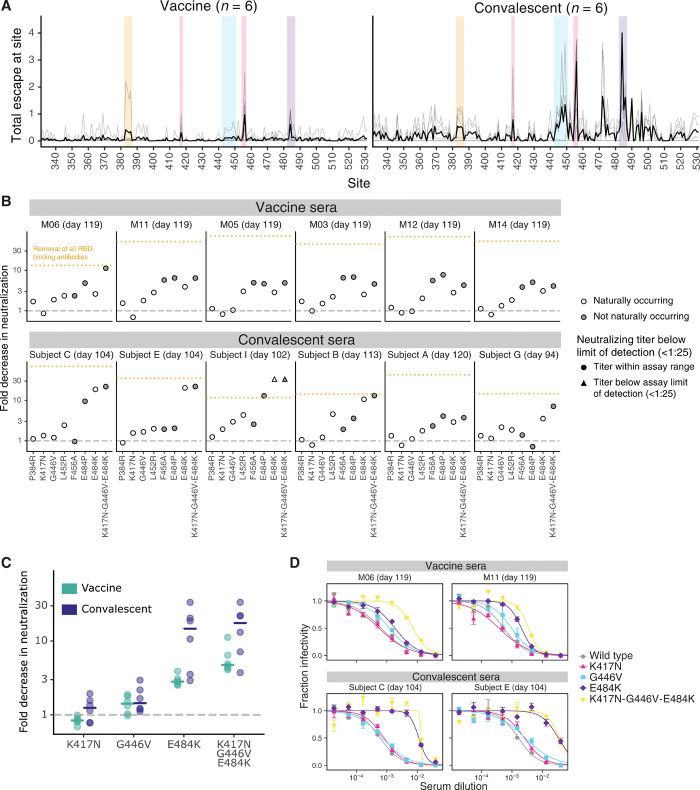
Effects of RBD mutations on neutralization by day 100 to 150 sera from vaccinated and convalescent individuals. (**A**) Total binding escape at each RBD site is shown for the samples from vaccinated (*n* = 6) or convalescent (*n* = 6) individuals tested in neutralization assays. The thin gray lines show individual samples, and the dark black line shows the mean. Key sites within each epitope are highlighted using the same color scheme as in Fig. 2A. (**B**) Neutralization of G614 spike-pseudotyped lentiviral particles with the indicated RBD mutations, shown as the fold decrease in NT_50_ compared to G614 spike with no additional mutations. Mutations that have been observed in human SARS-CoV-2 isolates are colored white, and nonnaturally occurring mutations are in gray. The orange dashed line represents the effect of depleting all RBD-binding antibodies. (**C**) The fold decrease in neutralization titer caused by individual mutations in each of the three major neutralizing epitopes of the RBD: K417 in the class 1 epitope, E484K in the class 2 epitope, and G446V in the class 3 epitope. The combination of all three mutations is also shown. Horizontal lines represent the median. In (B) and (C), the dashed gray line indicates no change in neutralization relative to unmutated spike. (**D**) Representative neutralization curves from two vaccine and two convalescent samples against the triple mutant and its composite single mutations.

We tested key RBD mutations in spike-pseudotyped lentiviral neutralization assays against a subset of vaccine and convalescent sera. We used the binding-escape maps to choose six representative samples each from the day 100 to 150 vaccine and convalescent sera for which >90% of the neutralizing activity was due to RBD-binding antibodies (Fig. 1 and fig. S1) (*15*). The escape maps for the vaccine and convalescent samples chosen for these assays are summarized in Fig. 5A and detailed in Fig. 2 and fig. S7.

We performed neutralization assays on mutants in each of the four major RBD epitopes (class 1, K417N and F456A; class 2, E484P and E484K; class 3, G446V and L452R; and class 4, P384R). Among these mutations, K417N, L452R, and E484K are present in emerging viral lineages, including B.1.351, P.1, B.1.427/429, B.1.526, and B.1.617 (*30*–*35*) that have been shown to have reduced neutralization (*3*, *5*–*8*, *36*, *37*). We also tested a triple mutant, K417N-G446V-E484K, with mutations in the class 1, 2, and 3 epitopes. For many convalescent sera, single RBD mutations reduced neutralization by approximately the same amount as removing all RBD-binding antibodies (Fig. 5B, figs. S8 and S9, and data file S3). However, no single RBD mutation we tested had a comparably large effect on vaccine sera (Fig. 5B). This result is consistent with the binding-escape maps, which generally indicate that vaccine sera have a broader RBD-binding specificity than convalescent sera.

The mutations that most affected neutralization also differed between vaccine and convalescent sera (Fig. 5B). For convalescent sera, the largest reduction in neutralization was consistently caused by mutations to site E484 in the class 2 epitope (*16*, *22*), including the E484K mutation present in multiple emerging viral lineages (*30*, *33*, *35*). In contrast, E484K generally caused a more moderate decrease in neutralization for vaccine sera. For some vaccine sera, another mutation at site E484 (E484P) caused a larger loss of neutralization, but E484P has not been found in any sequenced isolates of SARS-CoV-2 and has been shown to reduce both ACE2 binding affinity (*23*) and viral entry titers (fig. S8D). The F456A mutation to the class 1 epitope often reduced neutralization by vaccine sera, although it had little effect on convalescent sera; this mutation is also not observed in natural sequences and reduces viral entry titers (fig. S8D). Mutations to the class 3 epitope (G446V and L452R) modestly reduced neutralization by some vaccine and convalescent sera (Fig. 5B). However, P384R in the less-neutralizing core RBD class 4 epitope (*17*, *18*, *25*, *26*) and K417N in the class 1 epitope had little effect on neutralization by any sera, consistent with previous reports (*5*–*7*, *38*). Although single mutations sometimes caused large decreases in neutralization by convalescent sera, in no case did they reduce neutralization by vaccine sera >10-fold or to a titer <100 (Fig. 5B and fig. S8).

The fact that single mutations ablated the anti-RBD neutralizing activity of some convalescent sera, but only modestly eroded the activity of vaccine sera, suggests that the vaccine elicits neutralizing antibodies with a greater number of RBD specificities. To test this idea, we performed neutralization assays with a triple mutant (K417N-G446V-E484K) containing a mutation in each of the class 1, 2, and 3 epitopes. For convalescent sera, the E484K mutation alone often caused a decrease in neutralization comparable to the triple mutant (Fig. 5, C and D, and fig. S8), consistent with the convalescent escape maps showing a strong focus on site E484. In contrast, for vaccine sera, the triple mutant always reduced neutralization more than any of its constituent single mutations (Fig. 5, C and D, and fig. S8). Moreover, the triple mutant decreased neutralization to the same extent as removing all RBD-binding antibodies for only one of the six vaccine sera samples tested (Fig. 5B), indicating that the vaccine usually induces some neutralizing antibodies not escaped by mutations to sites K417, G446, and E484. These results are consistent with the escape maps indicating that the vaccine sera often have a broader RBD-binding specificity. Infection also elicited very broad anti-RBD neutralizing activity in some cases; for instance, serum from the convalescent individual with the broadest escape map (participant G, day 94) was substantially more affected by the triple mutant than any of its constituent single mutants (Fig. 5B and figs. S7 and S8).

## DISCUSSION

In this study, we have shown differences in the specificity of polyclonal serum antibodies acquired by infection versus vaccination with mRNA-1273. The neutralizing activity of vaccine sera is more targeted to the RBD than for convalescent sera, with most vaccine sera losing all detectable neutralization at a 1:25 cutoff after depletion of RBD-directed antibodies. This fact is unexpected because the mRNA-1273 vaccine encodes the full spike ectodomain (*11*), and one conjectured benefit of full-spike versus RBD-only vaccines was elicitation of neutralizing antibodies targeting non-RBD subdomains.

At first glance, the RBD targeting of the vaccine sera neutralization might seem likely to increase susceptibility to viral mutations, but the rest of our results suggest that this may not be the case. Our comprehensive maps of how RBD mutations reduce serum antibody binding show that vaccine-elicited antibodies are usually less affected by any single RBD mutation than infection-elicited antibodies. Whereas infection-elicited RBD antibodies are often strongly focused on an epitope including site E484, vaccine-elicited antibodies bind more broadly across the RBD, including to the more conserved “core” regions. This broader binding makes neutralization by vaccine sera more resistant to mutations within the RBD. For instance, RBD-directed neutralization by convalescent sera was greatly reduced or even eliminated by a combination of key mutations at the three major epitopes in the RBD’s receptor-binding motif, but all vaccine sera that we tested retained substantial neutralization against this triple mutant. This result implies either that vaccination induces an antibody response more broadly distributed across the RBD surface or that the individual antibodies elicited by vaccination are more robust to these mutations (*39*, *40*). Our results are consistent with a recent study by Amanat *et al.* (*19*), which reported that several single RBD mutations reduce binding of serum from individuals vaccinated with the Pfizer mRNA vaccine less than for serum from previously infected individuals.

We found that the specificity of the mRNA-1273 vaccine–induced RBD-binding antibody response often narrows over time. In contrast, the infection-elicited RBD-binding antibody response often broadens over time (*15*, *39*). However, because the early vaccine-induced RBD-binding antibody response is so broad compared to that induced by infection, even with these contrasting dynamics, the vaccine response remains broader than the convalescent response at late time points of 3 to 4 months. In addition, the overall antibody response is more homogeneous for vaccinated than convalescent individuals. For instance, the RBD binding titers, neutralizing titers, amount of neutralization derived from RBD-binding antibodies, and effects of mutations on neutralization were more uniform for the vaccinated cohort than the convalescent cohort.

Our results do not explain why there are differences between the vaccine- and infection-elicited antibody responses, but we note two possibilities. First, the vaccine encodes a stabilized S-2P spike, which could present some epitopes in slightly different conformations and lead to less S1 shedding. Second, the vaccine is delivered in a two-dose schedule by an mRNA-lipid nanoparticle, which may lead to different kinetics of antigen presentation than viral infection (*41*, *42*). Another recent study suggests that mRNA vaccination elicits a different distribution of isotypes and fewer antibodies that cross-react to common-cold coronaviruses as compared to infection (*43*).

There are several limitations to our study. The vaccinated individuals in our study were relatively young (18 to 55 years) and healthy, whereas the convalescent individuals were older (23 to 76 years; median, 56) with a range of comorbidities (*13*). In addition, we did not examine effects of mutations or deletions to the N-terminal domain of the spike protein, which can also affect neutralization by vaccine sera (*7*). Our experiments assayed binding of antibodies to isolated RBD expressed by yeast, and so cannot capture mutational effects on trimer conformation or antibodies with quaternary epitopes (*24*). Last, the N-linked glycans on yeast-expressed proteins are more mannose-rich than those on mammalian-expressed proteins (*44*).

Despite these limitations, our results in conjunction with other recent studies (*19*) suggest that mRNA vaccines and infection elicit somewhat distinct anti-spike antibody responses. Therefore, it is important to differentiate antibody immunity acquired by different means when assessing the impact of viral evolution. Considerable effort is being expended to identify emerging antigenic variants of SARS-CoV-2 and determine which ones might evade immunity (*3*, *7*, *8*, *35*). Our findings suggest that the results could vary depending on the source of immunity. Furthermore, carefully characterizing and comparing the specificity of antibody immunity elicited by additional vaccine modalities could provide a basis for determining whether some vaccine responses will be more resistant to viral evolution.

## MATERIALS AND METHODS

### Study design

De-identified post-vaccination sera were obtained as secondary research samples from the National Institute of Allergy and Infectious Diseases–sponsored mRNA-1273 phase 1 clinical trial (NCT04283461) (*12*). We obtained samples from 14 individuals who received two 250-μg doses of the mRNA-1273 vaccine and 8 individuals who received two 100-μg doses. All individuals were between 18 and 55 years old. The study size was determined by the number of samples that were available from the phase 1 clinical trial and not based on any power calculations. Experiments described in this manuscript were not performed blinded. The samples were collected under the human subject approvals described in (*12*). Because of the de-identified nature of the samples, the work described in this paper was deemed nonhuman subjects research by the Fred Hutchinson Cancer Research Center Institutional Review Board.

Previously reported results from samples from two cohorts of SARS-CoV-2 convalescent individuals are reanalyzed here (*15*, *16*). One cohort of convalescent plasma samples were previously described (*13*, *15*) and collected as part of a prospective longitudinal cohort study of individuals with SARS-CoV-2 infection in Seattle, WA, between February and July 2020. The plasma samples from 17 individuals were examined here (8 of 17 females; age range, 23 to 76 years; mean, 51.6 years; median, 56 years). All data from this cohort, including the neutralization and RBD- and spike-binding activity of plasma samples before and after depletion of RBD-binding antibodies in Fig. 1 and RBD binding-escape maps in Fig. 4 and figs. S6B and S7, were previously reported (*15*) with the exception of neutralization assays in Fig. 5 and figs. S8 and S9, which were performed in this study. This work was approved by the University of Washington Institutional Review Board.

All data from the second cohort of plasma samples (*n* = 5), including the aggregated escape maps in Fig. 4, were previously reported (*16*) and are reanalyzed here. The plasma samples were originally collected 21 to 35 days after symptom onset as part of a prospective longitudinal cohort study of SARS-CoV-2 convalescent individuals in New York, NY, under the human subject approvals described in (*14*).

### RBD deep mutational scanning library

The yeast-display RBD mutant libraries were previously described (*22*, *23*). Briefly, duplicate mutant libraries were constructed in the spike RBD from SARS-CoV-2 (isolate Wuhan-Hu-1, GenBank accession number MN908947, residues N331-T531) and contain 3804 of the 3819 possible amino acid mutations, with >95% present as single mutants. Each RBD variant was linked to a unique 16-nucleotide (nt) barcode sequence to facilitate downstream sequencing. As previously described, libraries were sorted for RBD expression and ACE2 binding to eliminate RBD variants that are completely misfolded or nonfunctional, such as those lacking modest ACE2 binding affinity (*22*).

### FACS sorting of yeast libraries to select mutants with reduced binding by polyclonal post-vaccination sera

Serum mapping experiments were performed in biological duplicate using the independent mutant RBD libraries, similarly to that previously described for monoclonal antibodies (*22*) and exactly as previously described for polyclonal plasma samples (*15*). Briefly, mutant yeast libraries induced to express RBD were washed and incubated with serum at a range of dilutions for 1 hour at room temperature with gentle agitation. For each serum, we chose a subsaturating dilution such that the amount of fluorescent signal due to serum antibody binding to RBD was approximately equal across samples. The exact dilution used for each serum is given in table S1. After the serum incubations, the libraries were secondarily labeled for 1 hour with 1:100 fluorescein isothiocyanate–conjugated anti-MYC antibody (Immunology Consultants Laboratory, CYMC-45F) to label for RBD expression and 1:200 Alexa Fluor 647–conjugated goat anti-human-IgA + IgG + IgM (Jackson ImmunoResearch 109-605-064) to label for bound serum antibodies. A flow cytometric selection gate was drawn to capture 3 to 6% of the RBD mutants with the lowest amount of serum binding for their degree of RBD expression (figs. S2 and S3). We also measured what fraction of cells expressing unmutated RBD fell into this gate when stained with 1× and 0.1× the concentration of serum. For each sample, about 10 million RBD^+^ cells (range, 7.3 × 10^6^ to 1.4 × 10^7^ cells) were processed on the BD FACSAria II cell sorter, with between 3 × 10^5^ and 6 × 10^5^ plasma-escaped cells collected per sample (table S1). Antibody-escaped cells were grown overnight in synthetic defined medium with casamino acids [yeast nitrogen base (6.7 g/liter), casamino acids (5.0 g/liter), MES acid (1.065 g/liter), and 2% w/v dextrose] to expand cells before plasmid extraction.

### DNA extraction and Illumina sequencing

Plasmid samples were prepared from 30 optical density (OD) units [1.6 × 10^8^ colony-forming units (CFUs)] of preselection yeast populations and about 5 OD units (~3.2 × 10^7^ CFUs) of overnight cultures of serum-escaped cells (Zymoprep Yeast Plasmid Miniprep II) as previously described (*22*). The 16-nt barcode sequences identifying each RBD variant were amplified by polymerase chain reaction and prepared for Illumina sequencing as described in (*23*). Barcodes were sequenced on an Illumina HiSeq 2500 with 50–base pair single-end reads. To minimize noise from inadequate sequencing coverage, we ensured that each antibody-escape sample had at least 2.5× as many post-filtering sequencing counts as FACS-selected cells, and reference populations had at least 2.5 × 10^7^ post-filtering sequencing counts.

### Analysis of deep sequencing data to compute each mutation’s escape fraction

Escape fractions were computed as described in (*22*), with minor modifications as noted below. We used the dms_variants package (https://jbloomlab.github.io/dms_variants/, version 0.8.5) to process Illumina sequences into counts of each barcoded RBD variant in each presort and antibody-escape population using the barcode/RBD look-up table from (*23*). For each serum selection, we computed the escape fraction for each barcoded variant using the deep sequencing counts for each variant in the original and serum-escape populations and the total fraction of the library that escaped antibody binding via the formula provided in (*22*). These escape fractions represent the estimated fraction of cells expressing that specific variant that falls in the escape bin, such that a value of 0 means that the variant is always bound by serum and a value of 1 means that it always escapes serum binding. We then applied a computational filter to remove variants with low sequencing counts or highly deleterious mutations that might cause antibody escape simply by leading to poor expression of properly folded RBD on the yeast cell surface (*22*, *23*). Specifically, we removed variants that had (or contained mutations with) ACE2 binding scores < −2.35 or expression scores < −1, using the variant- and mutation-level deep mutational scanning scores from (*23*). Note that these filtering criteria are slightly more stringent than those used in (*22*) but are identical to those used in (*15*, *16*, *27*).

We next deconvolved variant-level escape scores into escape fraction estimates for single mutations using global epistasis models (*45*) implemented in the dms_variants package, as detailed at https://jbloomlab.github.io/dms_variants/dms_variants.globalepistasis.html and described in (*22*). The reported scores throughout the paper are the average across the libraries; these scores are also in data file S2. Correlations in final single-mutant escape scores are shown in fig. S4.

For plotting and analyses that required identifying RBD sites of “strong escape,” we considered a site to mediate strong escape if the total escape (sum of mutation-level escape fractions) for that site exceeded the median across sites by >5-fold, and was at least 5% of the maximum for any site. Full documentation of the computational analysis is at https://github.com/jbloomlab/SARS-CoV-2-RBD_MAP_Moderna and archived in the Zenodo code repository under doi 10.5281/zenodo.4741330.

### Generation of pseudotyped lentiviral particles

Human embryonic kidney (HEK) 293T [American Type Culture Collection (ATCC), CRL-3216] cells were used to generate SARS-CoV-2 spike-pseudotyped lentiviral particles and 293T-ACE2 cells [Biodefense and Emerging Infectious Research Resources Repository (BEI Resources), NR-52511] were used to titer the SARS-CoV-2 spike-pseudotyped lentiviral particles and to perform neutralization assays (see below). We used spike-pseudotyped lentiviral particles that were generated essentially as described in (*46*), using a codon-optimized SARS-CoV-2 spike from the Wuhan-Hu-1 strain that contains a 21–amino acid deletion at the end of the cytoplasmic tail (*13*) and the D614G mutation that is now predominant in human SARS-CoV-2 (*47*). The plasmid encoding this spike, HDM_Spikedelta21_D614G, is available from Addgene (no. 158762) and BEI Resources (NR-53765), and the full sequence is at www.addgene.org/158762. Point mutations were introduced into the RBD of this plasmid via site-directed mutagenesis. Therefore, all mutations tested in this paper are in the G614 background and are compared to a “wild-type” spike with G614.

To generate these spike-pseudotyped lentiviral particles (*46*), 6 × 10^5^ HEK-293T (ATCC CRL-3216) cells per well were seeded in six-well plates in 2 ml of D10 growth media [Dulbecco’s modified Eagle’s medium with 10% heat-inactivated fetal bovine serum, 2 mM l-glutamine, penicillin (100 U/ml), and streptomycin (100 μg/ml)]. Twenty-four hours later, cells were transfected using BioT transfection reagent (Bioland Scientific) with a Luciferase_IRES_ZsGreen backbone, Gag/Pol lentiviral helper plasmid (BEI Resources NR-52517), and wild-type or mutant SARS-CoV-2 spike plasmids. Media were changed to fresh D10 at 24 hours after transfection. At ~60 hours after transfection, viral supernatants were collected, filtered through a 0.45-μm surfactant-free cellulose acetate low protein-binding filter, and stored at −80°C.

### Titering of pseudotyped lentiviral particles

Titers of spike-pseudotyped lentiviral particles were determined as described in (*46*) with the following modifications. One hundred microliters of diluted spike-pseudotyped lentiviral particles was added to 1.25 × 10^4^ 293T-ACE2 cells (BEI Resources NR-52511), grown overnight in 50 μl of D10 growth media in a 96-well black-walled poly-l-lysine–coated plate (Greiner Bio-One, 655936). Relative luciferase units (RLUs) were measured 65 hours after infection (Promega Bright-Glo, E2620) in the infection plates with a black back-sticker (Thermo Fisher Scientific, NC9425162) added to minimize background. Titers were first estimated from the average of eight twofold serial dilutions of virus starting at 25 μl of virus in a total volume of 150 μl, performed in duplicate, and normalized to a wild-type D614G variant harvested on the same day. Quantitative titering was then performed at a single virus dilution, targeting 200,000 RLU per well. Values in fig. S8D are shown as average RLUs per microliter measured across 16 technical replicates at a single dilution.

### Neutralization assays

293T-ACE2 cells (BEI Resources NR-52511) were seeded at 1.25 × 10^4^ cells per well in 50 μl of D10 in poly-l-lysine–coated, black-walled, 96-well plates (Greiner 655930). Twenty-four hours later, pseudotyped lentivirus supernatants were diluted to ~200,000 RLU per well (determined by titering as described above and incubated with a range of dilutions of serum for 1 hour at 37°C). One hundred microliters of the virus-antibody mixture was then added to cells. At about 50 or 70 hours after infection, luciferase activity was measured using the Bright-Glo Luciferase Assay System (Promega, E2610). Fraction infectivity of each serum antibody-containing well was calculated relative to a “no-serum” well inoculated with the same initial viral supernatant (containing wild-type or mutant RBD) in the same row of the plate. We used the neutcurve package (https://jbloomlab.github.io/neutcurve version 0.5.2) to calculate the inhibitory concentration 50% (IC_50_) and the neutralization titer 50% (NT_50_), which is 1/IC_50_, of each serum against each virus by fitting a Hill curve with the bottom fixed at 0 and the top fixed at 1.

### Depletion of RBD-binding antibodies from polyclonal sera

Two rounds of sequential depletion of RBD-binding antibodies were performed for vaccine-elicited sera. Magnetic beads conjugated to the SARS-CoV-2 RBD (ACROBiosystems, MBS-K002) were prepared according to the manufacturer’s protocol. Beads were resuspended in ultrapure water at 1 mg of beads per milliliter and a magnet was used to wash the beads three times in phosphate-buffered saline (PBS) with 0.05% bovine serum albumin (BSA). Beads were then resuspended in PBS with 0.05% BSA at 1 mg of beads per milliliter. Beads (manufacturer-reported binding capacity of 10 to 40 μg/ml anti-RBD antibodies) were incubated with human sera at a 3:1 (beads:serum) ratio (150 μl beads + 50 μl serum), rotating overnight at 4°C. A magnet (MagnaRack Magnetic Separation Rack, Thermo Fisher Scientific, CS15000) was used to separate antibodies that bind RBD from the supernatant, and the supernatant (the post-RBD antibody depletion sample) was removed. A mock depletion (predepletion sample) was performed by adding 150 μl of PBS + 0.05% BSA and incubating rotating overnight at 4°C. A second round of depletion was then performed to ensure full depletion of RBD-binding antibodies. For the neutralization assays on these sera depleted of RBD-binding antibodies, the reported serum dilution is corrected for the dilution incurred by the depletion process.

### Measurement of serum binding to RBD or spike by enzyme-linked immunosorbent assay

The IgG enzyme-linked immunosorbent assays (ELISAs) for spike protein and RBD were conducted as previously described (*48*). Briefly, ELISA plates were coated with recombinant spike and RBD antigens described in (*48*) at 2 μg/ml. Five threefold serial dilutions of sera beginning at 1:500 were performed in PBS with 0.1% Tween with 1% Carnation nonfat dry milk. Dilution series of the “synthetic” sera composed of the anti-RBD antibody REGN10987 (*49*) or anti–N-terminal domain antibody 4A8 (*21*) and pooled prepandemic human serum from 2017 to 2018 (Gemini Biosciences; nos. 100–110, lot H86W03J; pooled from 75 donors) were performed such that the anti-spike antibody was present at a highest concentration of 0.25 μg/ml. Both antibodies were recombinantly produced by GenScript. The rREGN10987 is that used in (*27*), and the variable domain heavy- and light-chain sequences for r4A8 were obtained from GenBank GI 1864383732 and 1864383733 (*21*) and produced on a human IgG1 and IgK background, respectively. Prepandemic serum alone, without anti-RBD antibody depletion, was used as a negative control, averaged over two replicates. Secondary labeling was performed with goat anti-human IgG-Fc horseradish peroxidase (HRP) (1:3000; Bethyl Laboratories, A80-104P). Antibody binding was detected with TMB/E HRP substrate (Millipore Sigma, ES001) and 1 N HCl was used to stop the reaction. OD_450_ was read on a Tecan Infinite M1000 Pro plate reader. The area under the curve was calculated using the scikit-learn python package, version 0.23.2 (https://scikit-learn.org/stable/), as the area under the titration curve with the serial dilutions on a log-scale.

### Data visualization

The static logo plot visualizations of the escape maps in the paper figures were created using the dmslogo package (https://jbloomlab.github.io/dmslogo, version 0.6.2), and in all cases, the height of each letter indicates the escape fraction for that amino acid mutation calculated as described above. For each sample, the *y* axis is scaled to be the greatest of (i) the maximum site-wise escape metric observed for that sample, (ii) 20× the median site-wise escape fraction observed across all sites for that serum, or (iii) an absolute value of 1.0 (to appropriately scale samples that are not “noisy” but for which no mutation has a strong effect on antibody binding). Sites K417, L452, S477, E484, and N501 have been added to logo plots because of their frequencies among circulating viruses. The code that generates these logo plot visualizations is available at https://github.com/jbloomlab/SARS-CoV-2-RBD_MAP_Moderna/blob/main/results/summary/escape_profiles.md and archived in the Zenodo code repository (doi 10.5281/zenodo.4741330). In many of the visualizations, the RBD sites are categorized by epitope region (*24*) and colored accordingly. We define the class 1 epitope as residues 403 + 405 + 406 + 417 + 420 + 421 + 453 + 455-460 + 473-476 + 486 + 487 + 489 + 504, the class 2 epitope as residues 472 + 483-485 + 490-494, the class 3 epitope as residues 345 + 346 + 437-452 + 496 + 498-501, and the class 4 epitope as residues 365-372 + 382-386.

For the static structural visualizations in the paper figures, the RBD surface [Protein Data Bank (PDB) 6M0J, (*50*)] was colored by the site-wise escape metric at each site, with white indicating no escape and red scaled to be the same maximum used to scale the *y* axis in the logo plot escape maps, determined as described above. We created interactive structure-based visualizations of the escape maps using dms-view (*51*) that are available at https://jbloomlab.github.io/SARS-CoV-2-RBD_MAP_Moderna/. The logo plots in these escape maps can be colored according to the deep mutational scanning measurements of how mutations affect ACE2 binding or RBD expression as described above.

For the composite line plots shown in Fig. 4, the convalescent (days 15 to 60) group includes two independent cohorts of individuals, one recruited in New York, NY (*n* = 5) (*14*), and another recruited in Seattle, WA (*n* = 11) (*13*). The convalescent (days 100 to 150) group is from the longitudinal cohort recruited in Seattle, WA (*n* = 11). The escape maps for convalescent individuals were previously reported in (*15*, *16*). The mRNA-1273 (day 119) group includes individuals who were vaccinated with either the 100- or 250-μg vaccine dose (*n* = 8 and *n* = 14, respectively). The *y*-axis maximum is scaled to 1.1 times the maximum group mean site-total escape among all groups, so outlier points exceeding this value are not shown.

### Statistical analysis

The percentage of neutralizing activity of vaccine-elicited sera and convalescent plasma due to RBD-binding antibodies is plotted with the plotnine python package, version 0.7.1 (https://plotnine.readthedocs.io/en/stable/index.html), shown as a Tukey boxplot (middle line indicating median, box limits indicating interquartile range) with individual measurements overlaid as points. *P* values are from a log-rank test accounting for censoring, calculated with the lifelines python package, version 0.25.10 (https://lifelines.readthedocs.io/en/latest/).

## SUPPLEMENTARY MATERIALS

stm.sciencemag.org/cgi/content/full/13/600/eabi9915/DC1
Figs. S1 to S9 Table S1 Data files S1 to S3

## Appendix

View/request a protocol for this paper from *Bio-protocol*.
